# Machine learning based power control in cellular and cell-free massive MIMO systems

**DOI:** 10.1038/s41598-026-38685-3

**Published:** 2026-02-10

**Authors:** Neda Ahmadi, Gholamreza Akbarizadeh

**Affiliations:** 1 School of Computer Science and Mathematics, Kingston University, London, UK; 2 Department of Electrical Engineering, Faculty of Engineering, Shahid Chamran University of Ahvaz, Ahvaz, Iran

**Keywords:** Cell-free massive MIMO, Cellular, WMMSE, Power control, sum-SE maximization, Deep learning, Spectral efficiency, Electrical and electronic engineering, Engineering

## Abstract

Effective power control (PC) is essential for optimizing performance in large-scale multiple-input multiple-output (mMIMO) networks. Traditional methods such as the weighted minimum mean square error (WMMSE) algorithm offer reliable estimates but require substantial computational overhead This study examines PC in mMIMO systems, focusing on aggregate spectral efficiency (sum SE) and the per-user SE cumulative distribution function (CDF). This investigation explores the impact of factors such as the number of UEs, access points/base stations (APs/BSs), and deep neural network (DNN)-based PC implementations in both cellular (CL) and cell-free (CF) architectures. We introduce a new metric ($\:\varDelta\:\mathrm{A}\mathrm{U}\mathrm{C}$ ) the area between the per-user SE CDFs of the DNN-based PC and the WMMSE baseline - as a compact and interpretable measure of ML versus optimization performance under deployment scaling. To the best of our knowledge, this is the first paper to systematically apply this metric across both cellular and cell-free mMIMO architectures while varying AP/BS count, antenna count, user density, and dataset size. By combining this metric with RMSE, sum-rate change, and execution-time analysis (Figs. 1, 2, 3, 4, 5 and 6, Table 6), we provide prescriptive guidance on when DNN-based PC not only matches but also outperforms WMMSE in both performance and real-time latency, enabling practical deployment in dense and low-latency networks.

## Introduction

Massive multiple-input multiple-output (mMIMO) technology enhances wireless communication by using multiple antennas at each base station (BS) or access point (AP), enabling simultaneous service to many users^1^. This architecture provides flexibility and improved performance, as users can be served by any antenna within the coverage area. Cell-free (CF) mMIMO systems differ from traditional cellular setups by eliminating cell boundaries. They rely on centralized processing units (CPUs) to coordinate multiple APs that share time-frequency resources in time division duplex (TDD) mode^2^. While CF systems benefit from reduced shadow fading and shorter distances between users and APs, they require a wireless or optical fronthaul for AP-to-CPU connectivity. As future mobile networks aim for higher capacity, lower latency, and better quality of service, substantial improvements in wireless design are essential^3^.

**Fig. 1:**
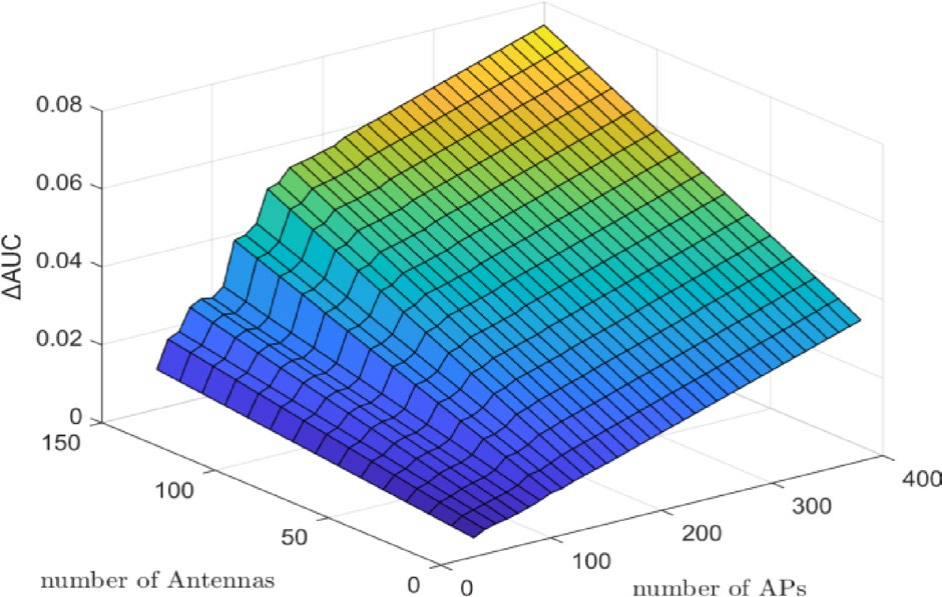
$\:\varDelta\:\mathrm{A}\mathrm{U}\mathrm{C}$ (bits/s/Hz) of DNN minus WMMSE for the CL architecture with $\:K\:=\:10$ UEs. The number of antennas per AP/BS $\:M$ varies from $\:10$ to $\:150$, and the number of APs $\:N\:$varies from $\:50$ to $\:400$. Results show that increasing $\:M$ and N gradually improves ∆AUC, indicating enhanced predictive performance of the DNN. Specifically, $\:\varDelta\:\mathrm{A}\mathrm{U}\mathrm{C}$ reaches $\:\sim0.03$ for $\:N\:=\:200$, $\:M\:=\:150$, and $\:\sim0.08$ for $\:N\:=\:400$, $\:M\:=\:200$, confirming that denser infrastructure improves DNN-based power control accuracy.

**Fig. 2:**
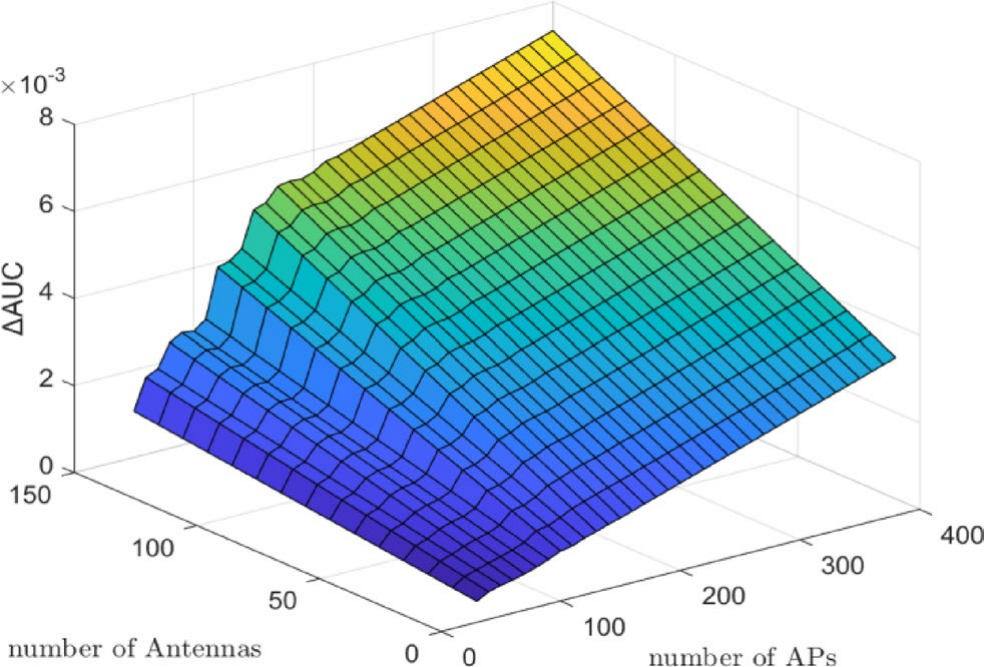
$\:\varDelta\:\mathrm{A}\mathrm{U}\mathrm{C}$ (bits/s/Hz) of DNN minus WMMSE for the CF architecture with $\:K\:=\:10$ UEs, $\:M\:=\:10-150\:$antennas, and $\:N\:=\:50-400$ APs. As $\:M$ and $\:N\:$increase, $\:\varDelta\:\mathrm{A}\mathrm{U}\mathrm{C}$ improves (up to $\:\sim0.008$ at $\:N\:=\:400$, $\:M\:=\:200$), demonstrating that CF systems benefit from additional antennas and APs. The results indicate the DNN’s superior ability to approximate optimal PC in distributed architectures.

**Fig. 3:**
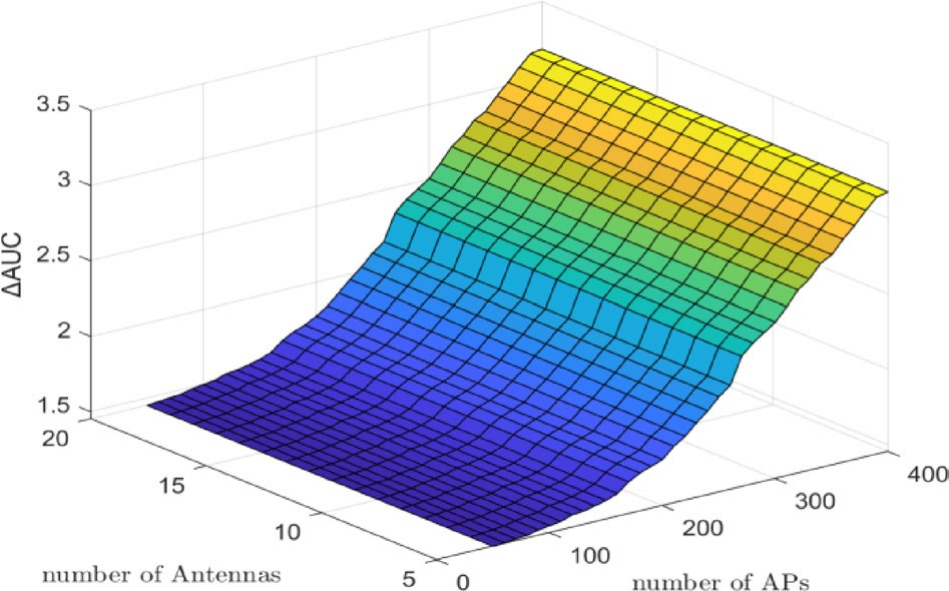
$\:\varDelta\:\mathrm{A}\mathrm{U}\mathrm{C}$ (bits/s/Hz) of DNN minus WMMSE for the CL architecture with $\:M\:=\:20$ antennas, $\:N\:=\:50-400$ APs, and varying $\:K\:=\:5-20$ UEs based on $\:\mathrm{100,000}$ samples. $\:\varDelta\:\mathrm{A}\mathrm{U}\mathrm{C}$ slightly decreases as $\:K$ increases, reflecting increased interference and complexity with higher user loads, but the DNN maintains robust performance, demonstrating scalability.

**Fig. 4:**
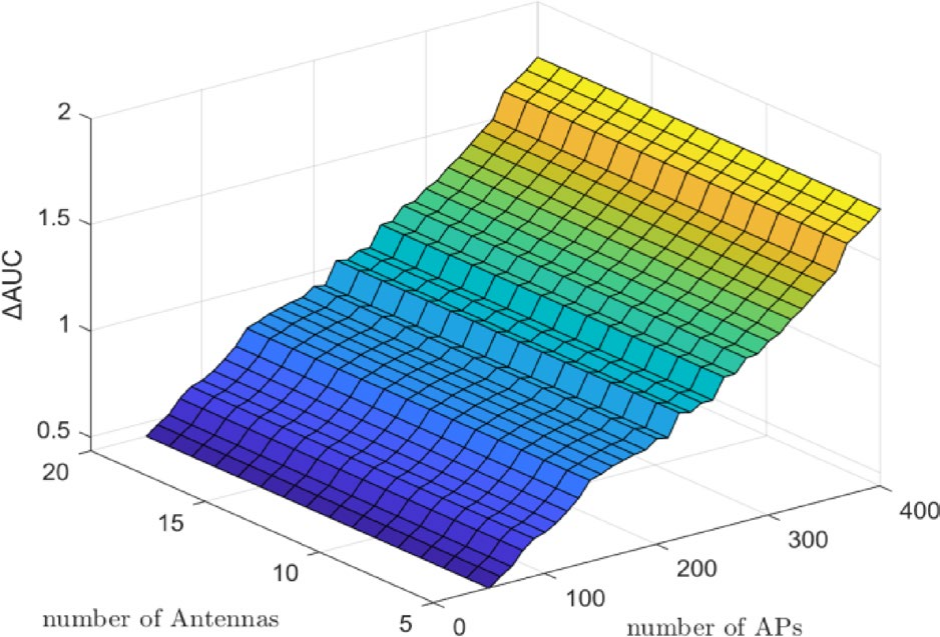
$\:\varDelta\:\mathrm{A}\mathrm{U}\mathrm{C}$ (bits/s/Hz) of DNN minus WMMSE for the CF architecture with $\:M\:=\:20$ antennas, $\:N\:=\:50-400$ APs, and $\:K\:=\:5-20\:$UEs based on $\:\mathrm{100,000}$ samples. $\:\varDelta\:\mathrm{A}\mathrm{U}\mathrm{C}$ rises with increasing training samples, indicating improved generalization of the DNN model and highlighting the importance of large datasets for reliable PC inference.

**Fig. 5:**
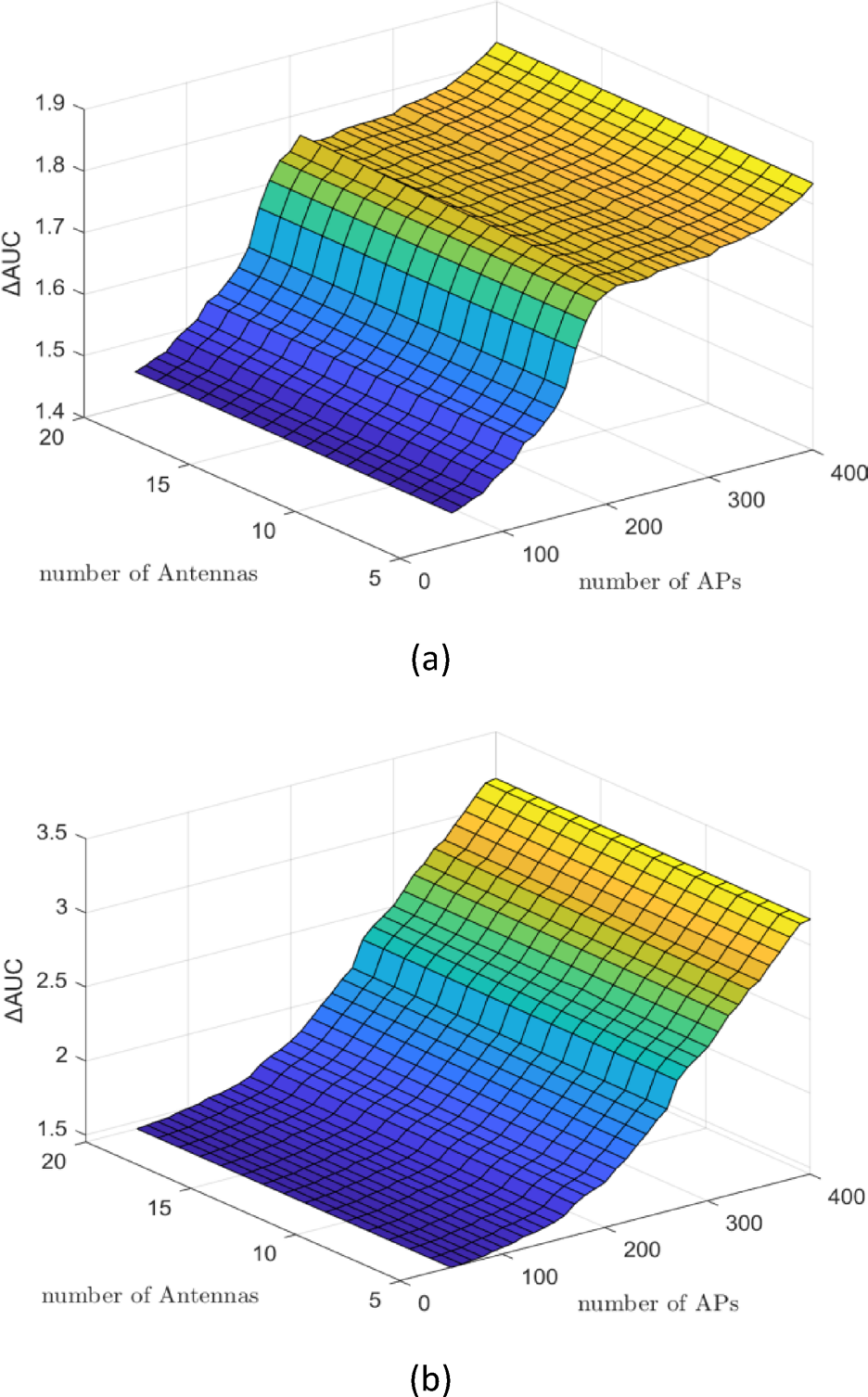
$\:\varDelta\:\mathrm{A}\mathrm{U}\mathrm{C}$ (bits/s/Hz) of DNN minus WMMSE for the CL architecture with $\:M\:=\:20$ antennas, $\:N\:=\:50-400$ APs, and $\:K\:=\:5-20$ UEs: (a) $\:\mathrm{50,000}$ training samples, (b) $\:\mathrm{100,000}\:$training samples. Increasing dataset size improves $\:\varDelta\:AUC$, confirming that larger datasets enhance DNN generalization and approximation of optimal PC.

**Fig. 6:**
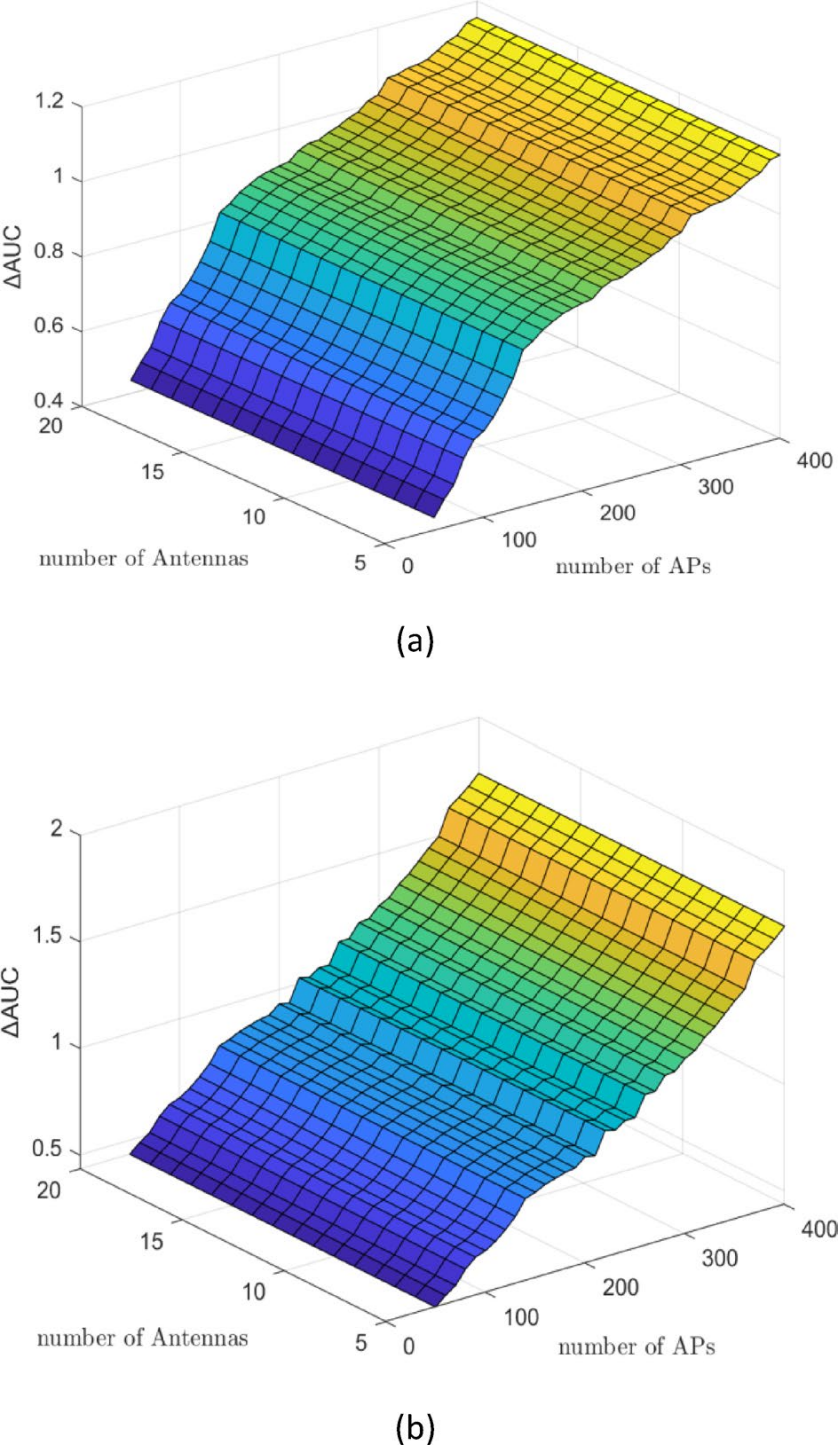
$\:\varDelta\:\mathrm{A}\mathrm{U}\mathrm{C}$ (bits/s/Hz) of DNN minus WMMSE for the CF architecture with $\:M\:=\:20$ antennas, $\:N\:=\:50-400$ APs, and $\:K\:=\:5-20$ UEs: (**a**) $\:\mathrm{50,000}$ training samples, (**b**) $\:\mathrm{100,000}$ training samples. Larger datasets lead to higher $\:\varDelta\:\mathrm{A}\mathrm{U}\mathrm{C}$, showing the DNN’s improved predictive performance in distributed CF networks, and confirming the model’s scalability.

PC optimization includes objectives such as maximizing the network’s weighted sum rate or minimizing power consumption, which makes it a complex problem^4^. Regardless of the objective, optimizing power allocation at BSs or APs reduces intertier and intracell interference, thereby improving network efficiency^5^. Despite its inherent challenges, PC remains crucial in mMIMO systems^6^. Unlike prior studies that focus mainly on traditional optimization or fixed-structure ML models, this work systematically analyzes how infrastructure scaling—specifically in the number of UEs, APs/BSs, and antennas—affects the performance of DNN-based PC algorithms compared to the WMMSE baseline. This paper makes four main contributions:

1. $\:\varDelta\:\mathrm{A}\mathrm{U}\mathrm{C}$ metric: We propose a robust metric that captures the area between per-user SE CDFs for DNN and WMMSE, enabling straightforward, distribution-aware comparison across systems and scales.
2. Large-scale, cross-architecture evaluation: We compare DNN and WMMSE across both CL and CF mMIMO architectures while systematically varying *N* (AP/BSs), *M* (antennas per AP), *K* (UEs), and dataset size ($\:50$*k* and $\:100$*k*), revealing trends not visible in single-topology studies (Figs. 1, 2, 3, 4, 5 and 6).
3. Input-representation analysis: We analyze how the channel representation vector g and input dimensionality influence DNN performance, explaining why infrastructure densification often improves DNN approximations of optimal PC.
4. Operational guidance: By combining this metric with RMSE and execution-time comparisons (Table 6), we provide practical recommendations on network densification and training data requirements to achieve both high SE and low inference latency for URLLC/6G scenarios.

These contributions extend prior ML-based PC research. For example^45^, improved model accuracy using ensembling but did not quantify system-level distributional gains or infrastructure scaling;^50^ demonstrated SVM/RBF competitiveness but did not explore deep architectures or large-sample scaling; and^54^ showed the feasibility of deep learning for power allocation but without cross-architecture analysis or latency comparisons as presented here. The main findings from our simulations are summarized in Sect. 5.

The subsequent sections of this paper are structured as follows: Sect. 2 reviews the pertinent literature on PC in massive MIMO systems, Sect. 3 elucidates the methodology employed, Sect. 4 delineates the experimental setup, Sect. 5 presents the experimental findings, and finally, Sect. 6 concludes the paper.

## Related works

### Exploring heuristic approaches for power control optimization

Several heuristic approaches have been proposed to address the PC problem in mMIMO systems^7–13^. Common techniques include WMMSE, successive convex approximation, and max-min fairness approaches^14–22^. Although effective, these methods often involve significant computational complexity, requiring numerous iterations and substantial processing resources to achieve convergence.

For example, one study^14^ focused on energy-efficient resource allocation (RA) using a two-stage approach. In the first stage, the WMMSE algorithm optimized beamforming vectors, while in the second stage, baseband unit (BBU) scheduling was handled using a bin-packing method based on the best-fit decreasing (BFD) algorithm. Another study^15^ employed successive convex approximation to maximize user energy efficiency (EE), taking into account transmit power, subcarrier allocation, and successive interference cancellation. The methodology also used Lagrangian optimization on GPUs and monotonic optimization to solve the problem efficiently.

The max-min fairness criterion has also been applied to optimize transmit power in various mMIMO systems^16–18^. For instance, in^19^, the max-min approach was used for power control in multicast CF mMIMO systems. Furthermore^20^, , introduced a framework based on fractional programming (FP) and sequential optimization to obtain a low-complexity suboptimal PC solution, evaluated in terms of energy efficiency (EE).

A dedicated iterative algorithm was proposed in^21^ to address the PC problem, with the objective of maximizing EE. The problem was reformulated as a one-dimensional quasiconcave optimization task, solved using the golden-section search method. In^23^, an efficient broadcast beamforming scheme based on pricing mechanisms was developed to maximize the weighted sum energy efficiency (WS-EE) in multi-input single-output (MISO) interference channels (ICs). Similarly^24^, presented an iterative method incorporating an approximation factor to evaluate the location-specific SE of distributed antenna systems (DASs). Additionally^25,26^, analyzed how PC parameters affect the performance of mobile device-to-device (D2D) cooperative communication systems.

Recent studies have also explored physical-layer security and ultra-reliable low-latency communication (URLLC) in CF-mMIMO networks. For example^27^, investigated rate-splitting multiple access (RSMA) to enhance physical-layer security in hardware-impaired CF-mMIMO systems. Studies^28] and [29^ examined URLLC performance under correlated Rician fading and RSMA strategies, providing insights into performance limits and optimization trade-offs. These contributions highlight the need for adaptive power control mechanisms and further motivate our DNN-based approach by emphasizing flexibility, scalability, and robustness for next-generation MIMO deployments.

### Harnessing machine learning for power control optimization

Prior studies have employed heuristic methods such as WMMSE, successive convex approximation, and max-min algorithms to manage power control complexity in mMIMO systems. However, these techniques are often limited by their high computational overhead, requiring substantial processing resources and multiple iterations to reach convergence^14–16,22,30,31^. As a result, recent research has explored artificial intelligence (AI) and ML techniques^32–38^ to alleviate the computational burden of PC problems.

Genetic algorithms have been examined as a heuristic solution to PC problems in wireless networks^14^. ML-based approaches for PC encompass a range of methods, including DNNs, deep reinforcement learning, the k-means algorithm with Gaussian mixture models, and the k-nearest neighbors algorithm^39–43^. For instance^18^, employed a deep learning (DL) model to address both sum-rate maximization and max-min PC in CF mMIMO systems. Additionally^44^, proposed an artificial neural network (ANN) model to approximate the WMMSE-based PC problem.

Another DNN-based approach^45^, incorporated noise as an input feature to enhance robustness against varying interference conditions. Similarly^39^, introduced a DNN-driven PC model for DASs, targeting joint optimization of SE and EE. In^46^ a DL approach was presented to solve the EE problem in the PC context, demonstrating reduced computational complexity across the network.

Similarly^47^, applied a DNN technique to multi-cell scenarios for optimized PC. In^48^ a CNN-based model was introduced to maximize either SE or EE, depending on system requirements. A DRL-based solution was presented in^49^ for spectrum sharing in cognitive radio networks, where the agent was trained to adjust transmit power and improve overall SINR. Recent studies in PC for CF mMIMO have also explored advanced optimization and access techniques to address hardware impairments, user density, and SE trade-offs. For instance, Ahmadi and Akbarizadeh^50^ proposed an SVM/RBF-based method for PC in both CL and CF mMIMO, demonstrating the potential of ML alternatives to traditional optimization frameworks.

Table 1 summarizes the abbreviations used throughout this paper for ease of reference.

**Table 1:** List of abbreviations used in the paper.

Abbreviate	Description
PC	Power control
mMIMO	Massive multiple-input-multiple-output
WMMSE	Weighted mean square error
ML	Machine learning
DL	Deep learning
SE	Spectral efficiency
CL	Cellular
CF	Cell-free
BS	Base station
AP	Access point
UE	User equipment
CPU	Centralized processing unit
TDD	Time division duplex
AUC	Area under the curve
RA	Resource allocation
BBU	Baseband unit
BFD	Best fit decreasing
EE	Energy efficiency
FP	Fractional programming
WS-EE	Weighted sum energy efficiency
MISO	Multi-input single-output
Interference channels	Interference channels
DAS	Distributed antenna systems
D2D	Device-to-device
AI	Artificial intelligence
ANN	Artificial neural network
DNN	Deep neural network
CNN	Convolutional neural network
DRL	Deep reinforcement learning
SINR	Signal-to-interference-plus-noise ratio
AWGN	Additive white Gaussian noise
MSE	Mean square error
ReLUs	Rectified linear units
CDF	Cumulative distribution function
URLLC	Ultra-reliable low-latency communications

## Methodology

This section outlines the methodology used to address the PC problem in mMIMO systems. While the WMMSE algorithm is a widely used baseline, it incurs significant computational complexity. Our work evaluates PC strategies in mMIMO systems using sum SE and the per-user SE CDF. This study systematically examines the effects of varying the number of UEs, APs/BSs, and deploying a DNN-based PC approach across both CL and CF architectures. Our experimental results highlight the influence of the parameter on the DNN input vector, as reflected in the $\:\varDelta\:\mathrm{A}\mathrm{U}\mathrm{C}$ (DNN minus WMMSE) metric. These findings highlight the importance of infrastructure scaling, particularly the number of APs/BSs and antennas, for achieving optimal PC performance in mMIMO systems.

## System model

### Cell-free architecture

Consider a CF mMIMO network in which multiple APs simultaneously serve UEs using the same time-frequency resources under a TDD protocol. The network includes $\:Z$ fronthaul links that connect all APs to a CPU. Each AP is equipped with antennas, while each UE has a single antenna. The channel gain vector between AP $\:n$ and UE $\:k\:$ is defined as follows:

1 $$\:{\mathbf{g}}_{n,k}\left(k\right)={\beta\:}_{n,k}^{\frac{1}{2}}{\mathbf{h}}_{n,k}$$

In this setup, the channel gain vector is characterized by two components. $\:{\beta\:}_{n,k}\ge\:0$ signifies the large-scale fading coefficient between AP $\:n$ (where $\:n\:=\:1,\:\dots\:,\:N)$ and UE $\:k\:$(where $\:k\:=\:1,\:\dots\:,\:K$). The small-scale fading is represented by the vector $\:{\mathbf{h}}_{n,k}\in\:{\mathbb{C}}^{M\times\:1}$, whose elements are independent and identically distributed complex Gaussian random variables with zero mean and unit variance, modeling uncorrelated Rayleigh fading. While this study adopts uncorrelated Rayleigh fading for simplicity, the proposed ML algorithm is flexible and can be extended to more general channel models, such as Rician fading, by retraining on appropriately generated data.

### Channel estimation

In the uplink, each AP estimates its local channel using uplink pilot signals transmitted by the UEs. We assume that local CSI is acquired independently at each AP through this pilot-based estimation process. The estimated CSI is then forwarded to the CPU for centralized processing and power control optimization. Channel estimation is performed using the minimum mean-squared error (MMSE) technique, resulting in the estimate $\:{\widehat{\mathbf{g}}}_{n,k}$, which consists of *M* independent Gaussian elements with identical statistical properties. The mean squared magnitude of the $\:m-th$ element is given as:

2 $$\:{\gamma\:}_{n,k}=\raisebox{1ex}{${\tau\:}_{p}{p}_{p}{\beta\:}_{n,k}^{2}$}\!\left/\:\!\raisebox{-1ex}{${\tau\:}_{p}{p}_{p}\sum\:_{{k}^{{\prime\:}}=1}^{K}{\beta\:}_{n,{k}^{{\prime\:}}}{\left|{\boldsymbol{\psi\:}}_{{k}^{{\prime\:}}}{\boldsymbol{\psi\:}}_{k}^{H}\right|}^{2}+1$}\right.$$

We consider a normalized pilot transmit power $\:{p}_{p}$ and a pilot sequence $\:{\boldsymbol{\psi\:}}_{k}$ for each UE *k*, where all sequences are pairwise orthogonal and satisfy the condition |$\:{\left|{\boldsymbol{\psi\:}}_{k}\right|}^{2}=1$. Let $\:{\tau\:}_{c}$ denote the coherence interval, within which a duration $\:{\tau\:}_{p}<{\tau\:}_{c}\:$is allocated for channel estimation, while the remaining $\:{\tau\:}_{c}-{\tau\:}_{p}$ symbols are used for downlink data transmission. Due to limited coherence time, the number of orthogonal pilot sequences may be fewer than the number of UEs, leading to pilot reuse across users. This results in pilot contamination - a critical issue in CF mMIMO systems - which degrades SE and limits system performance.

### Transmission of downlink data

Based on the acquired channel estimates, each AP employs normalized conjugate beamforming (NCB) to transmit signals to the intended UEs. Let $\:{q}_{k}$ denote the data symbol intended for user $\:k$, satisfying $\:\mathbb{E}\left\{{\left|{q}_{k}\right|}^{2}\right\}=1.$ The downlink signal transmitted from AP $\:n$, denoted as $\:{\mathbf{x}}_{n}$, is expressed as:

3 $$\:{\mathbf{x}}_{n} = \sum\limits_{{k^{\prime } = 1}}^{K} {\sqrt {p_{{n,k^{\prime } }} } \frac{{\widehat{{\mathbf{g}}}_{{n,k^{\prime } }} }}{{\sqrt {{\mathbb{E}}\left\{ {\left\| {\widehat{{\mathbf{g}}}_{{n,k^{\prime } }} } \right\|^{2} } \right\}} }}q_{{k^{\prime } }} } = \sum\limits_{{k^{\prime } = 1}}^{K} {\sqrt {p_{{n,k^{\prime } }} } \frac{{\widehat{{\mathbf{g}}}_{{n,k^{\prime } }} }}{{\sqrt {M\gamma _{{n,k^{\prime } }} } }}q_{{k^{\prime } }} }$$

Let $\:{p}_{n,{k}^{{\prime\:}}}$ denote the downlink transmit power from AP $\:n$ to user $\:{k}^{{\prime\:}}$, constrained by $\:{p}_{n,{k}^{{\prime\:}}}\le\:{p}_{\mathrm{m}\mathrm{a}\mathrm{x}}$, where $\:{p}_{\mathrm{m}\mathrm{a}\mathrm{x}}\:$represents the maximum allowable transmit power. The signal received by user $\:k$, denoted by $\:{y}_{k}$, is a superposition of the signals transmitted from all APs in the network and is expressed as follows:

4 $$\:{y}_{k}={\sum\:}_{n=1}^{N}{\sum\:}_{{k}^{{\prime\:}}=1}^{K}\sqrt{{p}_{n,{k}^{{\prime\:}}}}\frac{{{\mathbf{g}}_{n,k}^{T}\widehat{\mathbf{g}}}_{n,{k}^{{\prime\:}}}}{\sqrt{M{\gamma\:}_{n,{k}^{{\prime\:}}}}}{q}_{{k}^{{\prime\:}}}+{w}_{k}\:\:\:\:$$

where the additive noise at UE $\:k$ is denoted by $\:{w}_{k}\sim\mathcal{C}\mathcal{N}\left(\mathrm{0,1}\right)$.

### Spectral efficiency (SE)

The downlink SE for user $\:k$, denoted by $\:{SE}_{k}$ is given by:

5 $$\:{SE}_{k}=\left(1-\raisebox{1ex}{${\tau\:}_{p}$}\!\left/\:\!\raisebox{-1ex}{${\tau\:}_{c}$}\right.\right){\mathrm{l}\mathrm{o}\mathrm{g}}_{2}\left(1+\frac{M{\left(\sum\:_{n=1}^{N}\sqrt{{p}_{n,k}{\gamma\:}_{n,k}}\right)}^{2}}{M\sum\:_{{k}^{{\prime\:}}\ne\:k}{\left(\sum\:_{n=1}^{N}\sqrt{{p}_{n,{k}^{{\prime\:}}}{\gamma\:}_{n,{k}^{{\prime\:}}}}\raisebox{1ex}{${\beta\:}_{n,k}$}\!\left/\:\!\raisebox{-1ex}{${\beta\:}_{n,{k}^{{\prime\:}}}$}\right.\right)}^{2}{\left|{\psi\:}_{k}{\psi\:}_{{k}^{{\prime\:}}}^{H}\right|}^{2}+{\sum\:}_{{k}^{{\prime\:}}=1}^{K=1}{\sum\:}_{n=1}^{N}{p}_{n,{k}^{{\prime\:}}}{\beta\:}_{n,k}+1}\right)$$

### Maximization of sum-SE PC

The PC problem for maximizing the sum-SE is formulated as follows:

6 $$\begin{gathered} \:{\mathrm{max}}_{{p_{{n,k}} }} \sum\limits_{{k = 1}}^{K} {{\mathrm{SE}}_{k} } \hfill \\ \:s.t.\:p_{{n,k}} \le p_{{{\mathrm{max}}}} ,\quad \forall _{{n,k}} \hfill \\ \end{gathered}$$

### WMMSE method for power control in mMIMO systems

The PC problem can be addressed using heuristic algorithm such as WMMSE^51^, max-min fairness^16^, and fractional programming^52^. For instance, the WMMSE algorithm estimates the allocated transmit power $\:{P}_{n,k}$ based on the channel gain vector $\:{\mathbf{g}}_{n,k}$.

7 $$\:{P}_{n,k}=D\left({\mathbf{g}}_{n,k}\right)$$

The maximization problem described in Eq. (6) is nonconvex, and its computational complexity increases exponentially with the number of APs $\:\left(N\right)$ and users $\:\left(K\right).$ A widely adopted approach to address this challenge is the WMMSE algorithm^51,53^, which transforms the original sum-SE maximization problem into a weighted mean square error (MSE) minimization problem^8^. Problems (6) and (8) are mathematically equivalent under a known transformation that exploits the relationship between data rate and MSE. Specifically, the WMMSE framework reformulates the nonconvex sum-rate maximization objective as a tractable weighted MSE minimization, which can be solved iteratively. The WMMSE algorithm can be formulated as follows:

8 $$\begin{gathered} \:\mathop {{\mathrm{min}}}\limits_{{\{ \omega _{{n,k}} ,\mu _{{n,k}} ,\upsilon _{{n,k}} \} _{{n = 1,k = 1}}^{{N,K}} }} \sum\limits_{n}^{{N = 1}} {\sum\limits_{{k = 1}}^{K} {\alpha _{{n,k}} (\omega _{{n,k}} e_{{n,k}} - {\mathrm{log}}(\omega _{{n,k}} ))} } \hfill \\ \:s.t.\quad 0 \le \upsilon _{{n,k}} \le \sqrt {P_{{n,k}}^{{DL}} } ,n = 1, \ldots ,N,k = 1, \ldots ,K \hfill \\ \end{gathered}$$

In the WMMSE formulation, the optimization variables $\:{\omega\:}_{n,k},{\mu\:}_{n,k}$ and $\:{\upsilon\:}_{n,k}$ are real valued scalars. The parameter $\:{\alpha\:}_{n,k}$ denotes the priority weight assigned to the communication link between AP *n* and user *k*, while $\:{\omega\:}_{n,k}$ specifies a positive weight associated with the MSE term. The variables {$\:{\mu\:}_{n,k}$, $\:{\upsilon\:}_{n,k}\in\:\mathbb{R}$ } correspond to the transmit and receive beamforming coefficients. The symbol $\:{e}_{n,k}$ denotes the MSE, which is defined as follows:

9 $$\:{e}_{n,k}=\left({\mu\:}_{k}\right|{h}_{kk}|{\upsilon\:}_{k}-1{)}^{2}+\cdots\:+{\sum\:}_{n\ne\:k}\left({\mu\:}_{n}\right|{h}_{nk}|{\upsilon\:}_{n}{)}^{2}+{\sigma\:}_{n,k}^{2}{\mu\:}_{n,k}^{2}$$

To improve the sum-SE, the WMMSE algorithm iteratively searches for a local optimum by updating one of the variables $\:{\mu\:}_{n,k}$, $\:{\omega\:}_{n,k}$, or $\upsilon _{{n,k}}$ at each iteration *t*, while keeping the other two fixed. The value of $\:{\mu\:}_{n,k}$ is updated first based on the current values of $\:\left\{{\omega\:}_{n,k},{\upsilon\:}_{n,k}\right\}$. The details of this iterative procedure for the CF system are outlined in Algorithm 1. The algorithm terminates when the convergence criterion $\:{\omega\:}_{n,k}<\epsilon\:$ is met, where $\:\epsilon\:$ is a predefined threshold that governs the stopping condition. In this context, $\:{h}_{kk}\in\:\mathbb{C}$ represents the direct channel between transmitter $\:k$ and receiver $\:k$, $\:{h}_{nk}\in\:\mathbb{C}$ denotes the interference channel from transmitter $\:n$ to receiver $\:k$, and $\:{\sigma\:}_{n,k}^{2}$ is the noise power associated with AP *n* and user $\:k$.

**Algorithm 1:**
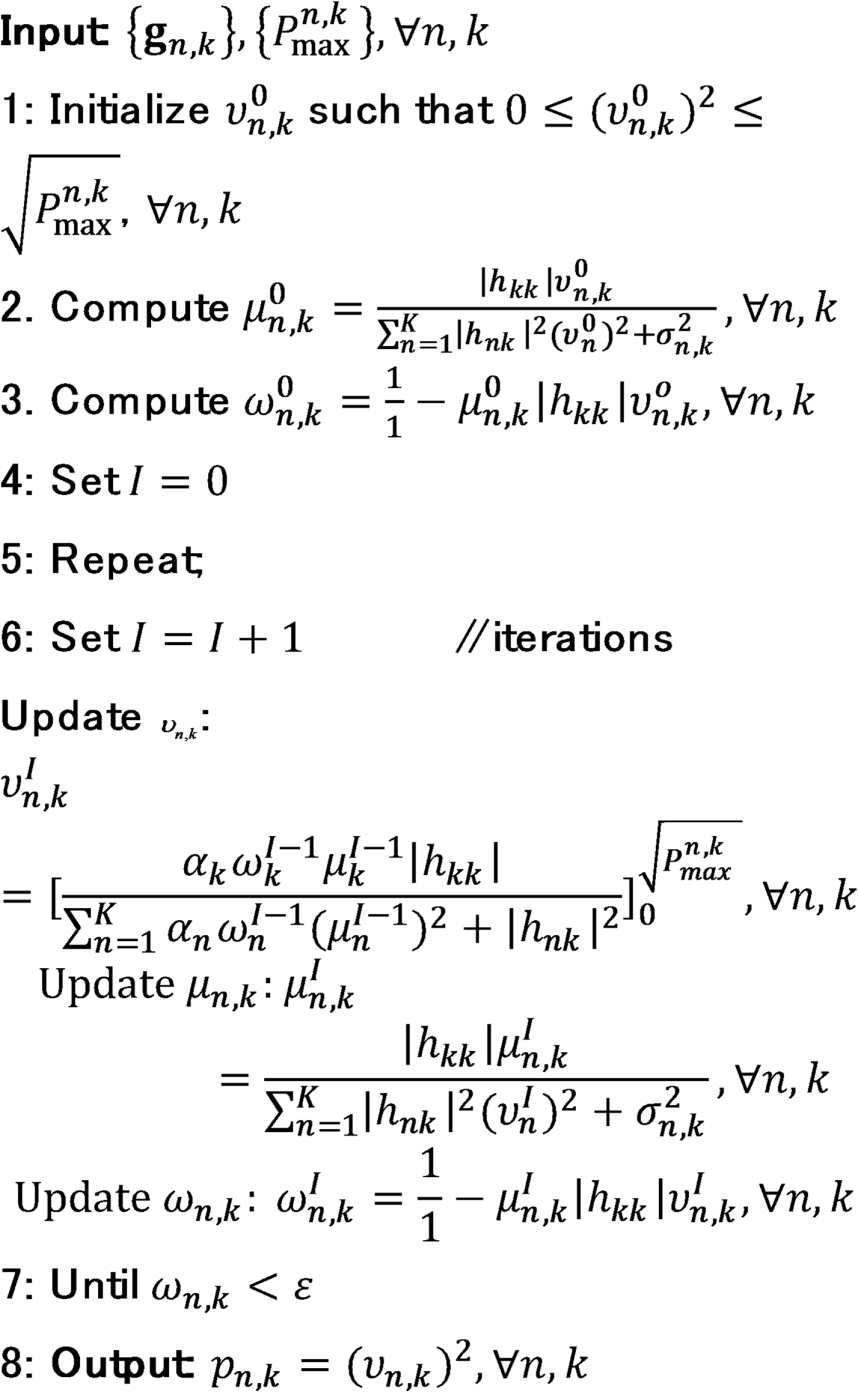
Pseudo code of WMMSE algorithm for CF system.

### DNN method for power control in mMIMO systems

Given the high computational complexity of the WMMSE algorithm, ML-based regression models can serve as efficient alternatives for PC. In this approach, the allocated power is approximated by $\:{\stackrel{\sim}{p}}_{n,k}$, defined as the output of a regression function $\:f$.

10 $$\:{\stackrel{\sim}{p}}_{n,k}=f\left({\mathbf{g}}_{n,k}\right)$$

with $$\:{\stackrel{\sim}{p}}_{n,k}\approx\:{p}_{n,k}$$.

11 $$\:{\mathbf{g}}_{k}=\left[{\mathbf{g}}_{1,k},\:\:\dots\:,\:\:{\mathbf{g}}_{N,k}\right]\in\:{\mathbb{C}}^{MN\times\:1}$$

### Cellular architecture

We consider the downlink of a single-cell mMIMO system, referred to as cell $\:l$, where a BS with $\:M$ antennas serves $\:K$ UEs over a shared time-frequency resource block. The channel gain vector from BS $\:l$ to UE $\:k$, associated with antenna $\:n$, is denoted by $\:{\mathbf{g}}_{lk}^{n}$.

12 $$\:{\mathbf{g}}_{lk}^{n}={\beta\:}_{lk}^{n}{\mathbf{h}}_{lk}^{n}$$

The large-scale fading coefficient between UE *k* in cell *l* and BS *n* is denoted as $\:{\beta\:}_{lk}^{n}$, where $\:{\beta\:}_{lk}^{n}$ $\:\ge\:0$. The small-scale fading vector is represented by $\:{\mathbf{h}}_{lk}^{n}\in\:{\mathbb{C}}^{M}$, whose elements are modeled as i.i.d. complex Gaussian random variables with zero mean and unit variance, corresponding to Rayleigh fading. More generally, the channel between BS $\:n$ and UE $\:k$ in cell $\:l$ can be modeled as $\:{\mathbf{h}}_{lk}^{n}\sim{\mathcal{N}}_{\mathbb{C}}\left({0}_{M},{\mathbf{R}}_{lk}^{n}\right)$, where $\:{\mathbf{R}}_{lk}^{n}\in\:{\mathbb{C}}^{M\times\:M}$ denotes the spatial correlation matrix, capturing correlated Rayleigh fading at the BS side.

### Channel estimation

In the uplink, the BS estimates the channels using pilot signals transmitted by the UEs. The estimation process uses MMSE estimation, resulting in an estimate $\:{\widehat{\mathbf{g}}}_{lk}^{n}$ which consists of $\:M$ independent Gaussian elements with identical statistical properties. The mean square magnitude of the $\:m-th$ element can be expressed as:

13 $$\:{\gamma\:}_{lk}^{n}=\frac{{\tau\:}_{p}{p}_{p}{\beta\:}_{lk}^{n}}{{\tau\:}_{p}{p}_{p}\sum\:_{{k}^{{\prime\:}}=1}^{K}{\beta\:}_{l{k}^{{\prime\:}}}^{n}{\left|{\boldsymbol{\psi\:}}_{{k}^{{\prime\:}}}{\boldsymbol{\psi\:}}_{k}^{H}\right|}^{2}+1}$$

We consider a normalized pilot power $\:{p}_{p}$ and a pilot sequence $\:{\boldsymbol{\psi\:}}_{k}$ consisting of binary $\:(0\:-\:1)\:$elements, with each sequence satisfying $\:{\left|{\boldsymbol{\psi\:}}_{k}\right|}^{2}=1$. Let $\:{\tau\:}_{c}$ denote the coherence time, with $\:{\tau\:}_{p}<{\tau\:}_{c}$ allocated for uplink channel estimation and the remaining $\:{\tau\:}_{c}-{\tau\:}_{p}$ symbols reserved for downlink data transmission. Ideally, if $\:{\tau\:}_{p}$ *≥ K*, each user could be assigned a unique orthogonal pilot sequence. However, in practical systems where coherence time is limited, we typically encounter the case $\:{\tau\:}_{c}$, $\:{\tau\:}_{p}<K$, which necessitates pilot reuse and may lead to pilot contamination.

### Downlink data transmission

Based on the estimated channel state information (CSI), the BS applies normalized conjugate beamforming (NCB) to transmit signals to the UEs. The beamforming vectors are computed using the estimated instantaneous CSI without applying any expectation operator, consistent with standard NCB practice. Let $\:{q}_{k}$ represent the data symbol intended for user $\:k$, satisfying $\:\mathbb{E}\left\{{\left|{q}_{k}\right|}^{2}\right\}=1$. The downlink signal transmitted from BS $\:n$, denoted by $\:{\mathbf{x}}_{n}\:$ is expressed as follows:

14 $$\:{x}_{l}^{n}={\sum\:}_{{k}^{{\prime\:}}=1}^{K}\sqrt{{p}_{l{k}^{{\prime\:}}}^{n}}\frac{{\widehat{\mathbf{g}}}_{l{k}^{{\prime\:}}}^{n}}{\sqrt{M{\gamma\:}_{l{k}^{{\prime\:}}}^{n}}}{q}_{{k}^{{\prime\:}}}$$

The beamforming vector is normalized by $\:\sqrt{M{\gamma\:}_{l{k}^{{\prime\:}}}^{n}}$ ​​, is derived from deterministic large-scale fading parameters. This formulation is consistent with standard normalized conjugate beamforming techniques, as presented in^27^. Following conventional practice, no expectation operator is applied during the normalization process. The downlink transmit power from BS $\:n$ to user $\:{k}^{{\prime\:}}$ is denoted by $\:{p}_{l{k}^{{\prime\:}}}^{n}$, subject to the constraint $\:{p}_{l{k}^{{\prime\:}}}^{n}\le\:{p}_{\mathrm{m}\mathrm{a}\mathrm{x}}$, where $\:{p}_{\mathrm{m}\mathrm{a}\mathrm{x}}$ is the maximum allowable transmit power. The received signal at user $\:k$ denoted by $\:{y}_{k}^{l}$, aggregates the contributions from all BSs in the network and is given by:

15 $$\:{y}_{lk}\:={\sum\:}_{n=1}^{N}{\sum\:}_{{k}^{{\prime\:}}=1}^{K}\sqrt{{p}_{l{k}^{{\prime\:}}}^{K}}\frac{{(\mathbf{g}}_{lk}^{n}{)}^{T}{\widehat{\mathbf{g}}}_{l{k}^{{\prime\:}}}^{n}}{\sqrt{M{\gamma\:}_{l{k}^{{\prime\:}}}^{n}}}{q}_{l{k}^{{\prime\:}}}+{w}_{lk}$$

The additive noise at user $\:k$ in cell $\:l$, denoted by $\:{w}_{lk}$, is modelled as a complex Gaussian random variable with zero mean and unit variance, i.e., $\:{w}_{lk}$ $\:\sim\mathcal{\:}\mathcal{C}\mathcal{N}\left(\mathrm{0,1}\right)$

### Spectral efficiency (SE)

The downlink SE for user $\:k$ in cell $\:l$ denoted by $\:{SE}_{lk}$, quantifies the data transmission efficiency in bits per second per hertz (bps/Hz).

16 $$\begin{gathered} \:SE_{{lk}} = \left( {1 - \tau _{p} /\tau _{c} } \right) \hfill \\ {\mathrm{log}}_{2} \left( {1 + \frac{{M\left( {\sum {_{{n = 1}}^{N} } \sqrt {p_{{lk}}^{n} \gamma _{{lk}}^{n} } } \right)^{2} }}{{M\sum {_{{k^{\prime } \ne k}} } \left( {\sum {_{{n = 1}}^{N} } \sqrt {p_{{lk^{\prime } }}^{n} \gamma _{{lk^{\prime } }}^{n} } \beta _{{lk}}^{n} /\beta _{{lk^{\prime } }}^{n} } \right)^{2} \left| {\psi _{k}^{l} \psi _{{k^{\prime } }}^{{lH}} } \right|^{2} + \sum\nolimits_{{k^{\prime } = 1}}^{{K = 1}} {\sum\nolimits_{{n = 1}}^{N} {p_{{lk^{\prime } }}^{n} \beta _{{lk}}^{n} + 1} } }}} \right) \hfill \\ \end{gathered}$$

### Maximization of sum-SE PC

The objective of maximizing the sum-SE through PC is formulated as follows:

17 $$\begin{gathered} \:{\mathrm{max}}_{{p_{{lk}}^{n} }} \sum\limits_{{k = 1}}^{K} {SE_{{lk}} } \hfill \\ \:s.t.\:p_{{lk}}^{n} \le \:p_{{{\mathrm{max}}}} ,\quad \forall _{{lk}}^{n} \hfill \\ \end{gathered}$$

### WMMSE method for power control in mMIMO systems

To solve the PC problem in the mMIMO system, we adopt the WMMSE algorithm. The allocated power $\:{p}_{lk}^{n}$ is estimated from the channel gain vector $\:{\mathbf{h}}_{lk}^{n}$, expressed as follows:

18 $$\:\:\:\:\:\:\:\:\:\:\:\:\:\:\:\:{p}_{lk}^{n}=D\left({\mathbf{h}}_{lk}^{n}\right)$$

The maximization problem in Eq. (17) is nonconvex, and its computational complexity grows exponentially with the number of APs $\:\left(N\right)$ and users $\:\left(K\right)$. To address this challenge, a widely adopted solution is the WMMSE algorithm^51,53^, which reformulates the original sum SE maximization problem into a tractable MSE minimization problem. The WMMSE formulation leverages the equivalence between rate maximization and MSE minimization, enabling iterative optimization of auxiliary variables under power constraints. The structure of the WMMSE algorithm is given as follows:

19 $$\:\underset{\{{\omega\:}_{lk}^{n},{\mu\:}_{lk}^{n},{\upsilon\:}_{lk}^{n}{\}}_{k=1,n=1}}{{\mathrm{m}\mathrm{i}\mathrm{n}}_{lk}^{n}}{\sum\:}_{n=1}^{N}{\sum\:}_{k=1}^{K}{\alpha\:}_{lk}^{n}({\omega\:}_{lk}^{n}{e}_{lk}^{n}-\mathrm{log}({\omega\:}_{lk}^{n}))$$

In the above formulation, the optimization variables $\:{\omega\:}_{lk}^{n},{\mu\:}_{lk}^{n}$ and $\:{\upsilon\:}_{lk}^{n}\:$ are real-valued scalars. The parameter $\:{\alpha\:}_{lk}^{n}$ denotes the priority assigned to the communication link between BS $\:n$ and UE $\:k$. The weights $\:{\omega\:}_{lk}^{n}\:$ are strictly positive, and the beamforming related coefficients {$\:{\mu\:}_{lk}^{n}$, $\:{\upsilon\:}_{lk}^{n}$} belong to the set of real numbers ($\:\mathbb{R}$). The term $\:{e}_{lk}^{n}\:$represents the MSE, which is defined as follows:

20 $$e_{{lk}}^{n} = (\mu _{{lk}}^{n} |h_{{lkk}}^{n} |\upsilon _{{lk}}^{n} - 1)^{2} + + \sum\limits_{{n \ne k}} {(\mu _{l}^{n} |h_{{lk}}^{n} |\upsilon _{l}^{n} )^{2} } + \quad (\sigma _{{lk}}^{n} )^{2} (\mu _{{lk}}^{n} )^{2}$$

To enhance the sum-SE, the WMMSE algorithm searches for local optimum by updating one of the three variables $\:{\mu\:}_{lk}^{n}$, $\:{\upsilon\:}_{lk}^{n}\:$and $\:{\omega\:}_{lk}^{n}$ at each iteration $\:t$, while keeping the others fixed. The optimal value of $\:{\mu\:}_{lk}^{n}\:$ is computed based on the current values of $\:\left\{{\omega\:}_{lk}^{n},{\upsilon\:}_{lk}^{n}\right\}$. The detailed steps of the WMMSE procedure for CL systems are outlined in Algorithm 2. The algorithm terminates when the convergence condition $\:{\omega\:}_{lk}^{n}<\epsilon\:$ is met, with as $\:\epsilon\:$ predefined threshold determined by the desired convergence behaviour. In this context, $\:{h}_{lkk}^{n}\in\:\mathbb{C}$ denotes the direct channel from transmitter $\:k$ to receiver $\:k$, $\:{h}_{lkk}^{n}\in\:\mathbb{C}$ denote the interference channel from transmitter *n* to receiver $\:k$, and $\:{(\sigma\:}_{lk}^{n}{)}^{2}\:$ represent the noise power at BS $\:n$ for UE $\:k$.

**Algorithm 2:**
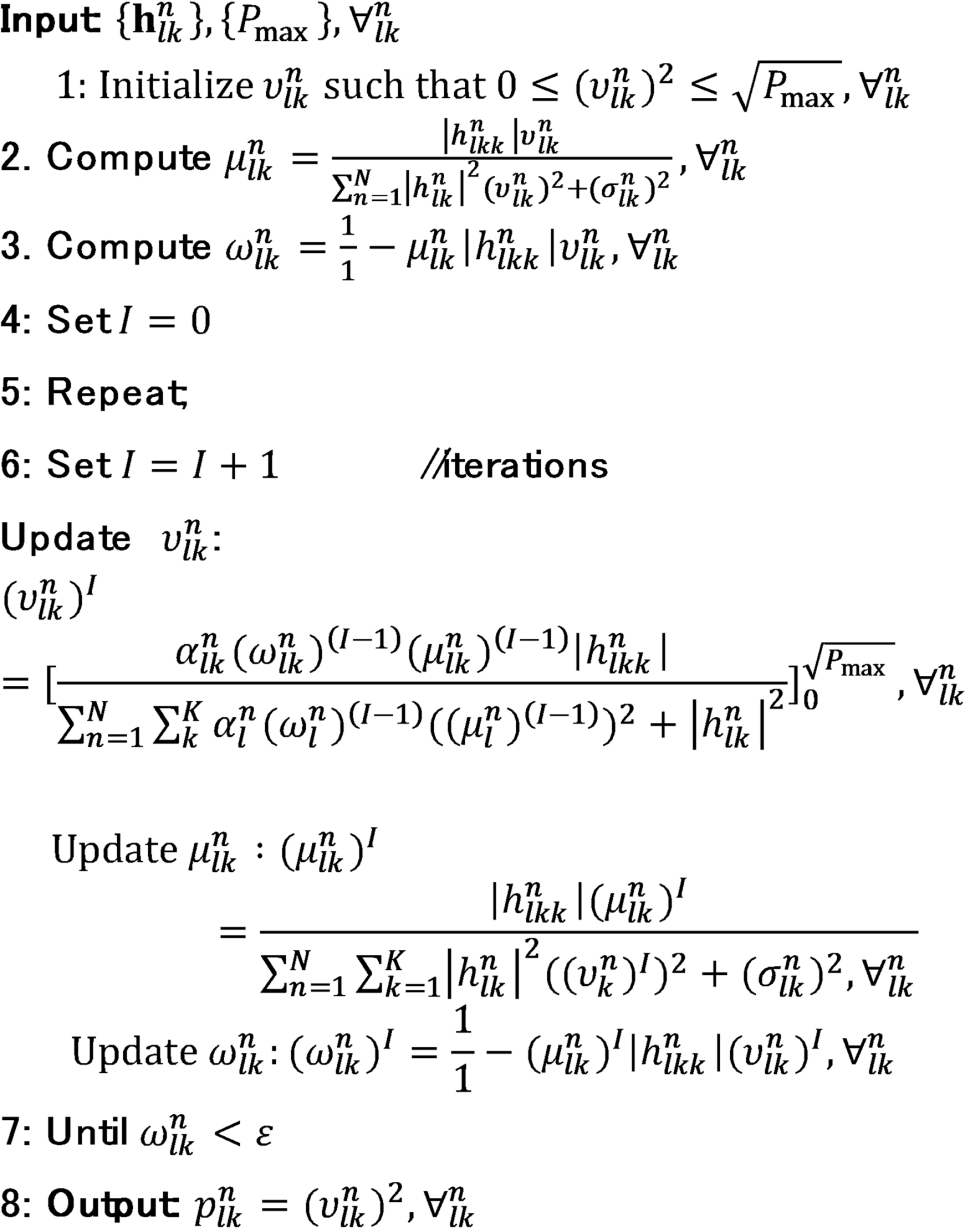
Pseudo code of the WMMSE algorithm for CL system.

### DNN method for power control in mMIMO systems

Due to its computational complexity of the WMMSE algorithm, real-time implementation in large-scale systems is challenging. As an alternative, ML-based regression models can be employed to approximate the WMMSE-based PC. In this approach, the estimated transmit power is denoted by $\:{\stackrel{\sim}{p}}_{lk}^{n}$, is defined as the output of a learned mapping function $\:f$.

21 $$\:{\stackrel{\sim}{p}}_{lk}^{n}=f\left({\mathbf{h}}_{lk}^{n}\right)$$

with $$\:{\stackrel{\sim}{p}}_{lk}^{n}\approx\:{p}_{lk}^{n}$$.

22 $$\:{\mathbf{h}}_{lk}=\left[{\mathbf{h}}_{lk}^{1},\:\:\dots\:,\:\:{\mathbf{h}}_{LK}^{N}\right]\in\:{\mathbb{C}}^{MN\times\:1}$$

## Experimental setup

This section presents the key components of our experimental framework, including the PC dataset and the ML algorithms used to evaluate regression-based PC benchmarks. The dataset was carefully constructed to reflect a wide range of scenarios and network configurations, capturing the fundamental characteristics of mMIMO systems. Machine learning models - specifically DNNs - were selected and fine-tuned to learn the complex, nonlinear relationships in the data, enabling accurate predictions for PC optimization.

### Datasets

To evaluate the proposed ML methods for PC, we considered both CL and CF mMIMO architectures, following the network configurations described in^54] and [55^, respectively. For both systems, the coverage area was set to $\:300\:m\:\times\:\:300\:m\:$operating at a carrier frequency of $\:3.4\:GHz$ and a bandwidth of $\:20\:MHz$. The APs/BSs were uniformly distributed within a $\:100\:m\:\times\:\:100\:m$ square area. UEs were randomly deployed across the entire coverage area, with a height difference of 5 m assumed between APs/BSs and UEs for accurate distance calculations. The noise variance was fixed at $\:{\sigma\:}^{2}\:=\:-\:95$ dBm, and the coherence time was set to $\:200$ modulation symbols, as in^2^. Uplink channel estimation was performed using pilot sequences of length $\:6$ symbols. We evaluated a range of infrastructure configurations, including the number of APs/BSs $\:(N\:=\:[50,\:60,\:\dots\:,\:\mathrm{a}\mathrm{n}\mathrm{d}\:400\left]\right)$ and UEs $\:(K\:=\:[5,\:6...,\:20\left]\right)$, each equipped with a single antenna. UE mobility was modelled by assigning each user a direction (up, down, left, or right) and a velocity uniformly distributed between $\:0\:$and $\:1\:m/s$. Each UE maintained a constant speed and direction for one second before randomly selecting new parameters. Initial UE positions at $\:t\:=\:0$ were uniformly distributed across the coverage region.

For each architecture, two datasets were generated: one with $\:{N}_{T}=\:\mathrm{50,000}$ samples and another with $\:{N}_{T}$ $\:=\:\mathrm{100,000}$ samples, corresponding to independent realizations of UE positions and channel states. Large-scale fading was modeled using the methodology described in^55^, incorporating both path loss and shadowing effects. The remaining network parameters used in the simulations were the same as those specified in^54^ for the CL network and^55^ for the CF-mMIMO network. The large-scale fading model includes log-normal shadowing with a standard deviation of 8 dB, and path loss is modeled with an exponent of $\:3.76$. The small-scale fading component follows an i.i.d. Rayleigh distribution. All users and APs/BSs were randomly deployed within a $\:1\:km\:\times\:\:1\:km$ area, and channel realizations were generated using Monte Carlo simulations over multiple drops. A summary of the key simulation parameters is provided in Table 2.

### Data preprocessing and training/validation split

Prior to training, all channel coefficients were normalized to zero mean and unit variance to ensure stable convergence. Outliers arising from anomalous channel realizations were clipped at ± 3 standard deviations. In addition, all input features were rescaled to the [0, 1] range when using activation functions sensitive to input scale (e.g., ReLU). For each dataset configuration (CL and CF, with varying numbers of APs/BSs, antennas, users, and dataset sizes), the data were randomly partitioned into 80% training and 20% validation subsets. This procedure was applied independently to each dataset to avoid any overlap between training and validation samples.

**Table 2:** Simulation parameters.

Parameters	Value
The CL/CF radius	$\:300\:m\:\times\:\:300\:m$
Carrier frequency	$\:3.4\:GHz$
Bandwidth	$\:20\:MHz$
APs/BSs radius	$\:100\:m\:\times\:\:100\:m$
Noise variance$\:\:{\sigma\:}^{2}$	$\:-95\:dBm$
Coherence time	$\:200$
Length of the uplink pilot	$\:6$
APs/BSs $\:N$	$\:[50,\:60,\:\dots\:,\:400]$
Number of users $\:K$	$\:[5,\:6,\:\dots\:,\:20]$
Velocity regarding the movement of each UE	$\:0\:\mathrm{a}\mathrm{n}\mathrm{d}\:1\:m/s$
Dataset samples $\:{N}_{T}$	$\:\mathrm{50,000}\:\mathrm{a}\mathrm{n}\mathrm{d}\:\mathrm{100,000}$

### Deep neural network (DNN) algorithms

The DNN algorithm is used to approximate the action-value function through a feedforward neural network comprising seven fully connected hidden layers. The number of neurons in each hidden layer is configured as $\:512,\:256,\:128,\:\:64,\:32,\:16,\:\mathrm{a}\mathrm{n}\mathrm{d}\:10$. The first hidden layer uses the exponential linear unit (ELU) activation function to support faster convergence and improved learning stability. The second through sixth layers use the rectified linear unit (ReLU), which outputs zero for negative inputs and equals the input otherwise. The final hidden layer applies the hyperbolic tangent (tanh) activation, while the output layer uses a linear activation function to accommodate the regression objective. The model is trained using the Adam optimizer for weight updates $\:\theta\:$, with a mini-batch size of $\:256$, following the setup described in^45^. For both the CL and CF-mMIMO architectures, the DNN takes an input of dimension $\:N\:\times\:\:K$, where $\:N\:=\:36$ and $\:K\:=\:10$.

Table 3 summarizes the training parameters used in our experiments, while Table 5 outlines the architecture of the DNN models. The DNN architecture was selected based on empirical analysis, and prior guidelines^54^. Its layered structure progressively reduces input dimensionality while enabling the network to learn complex nonlinear relationships, a design shown effective in high-dimensional wireless communication scenarios. The first hidden layer employs the exponential linear unit (ELU) activation function to facilitate smoother optimization and faster convergence. ReLU (rectified linear unit) activations are applied in the middle layers to address the vanishing gradient issue and support stable learning. The final output layer uses a linear activation function, which is well suited for regression-based tasks such as predicting PC coefficients. This configuration strikes a practical balance between model complexity and predictive performance and was validated through extensive empirical testing.

**Table 3:** Training parameters for DNN models.

Symbols	Values
Learning rate	$\:0.005$
Batch size	$\:200$
Epochs	$\:100$
Optimizer	Adam

### Cross-validation and generalization assessment

To confirm the robustness of our results, we performed five independent training runs with different random 80/20 splits and reported the averaged metrics (ΔAUC, RMSE, and execution time). While we did not employ full k-fold cross-validation due to the computational cost of training at this scale, the repeated random splits provided a reliable measure of generalization consistency.

### Hyperparameter selection

The DNN architecture (number of hidden layers, neurons per layer, activation functions, optimizer, and learning rate) was determined via a grid search on the validation set. The selected configuration - three hidden layers with 256, 128, and 64 neurons, ReLU activation, the Adam optimizer, and a learning rate of 0.001 - provided the best balance between predictive accuracy and inference latency. The final set of hyperparameters used in all experiments is summarized in Table 4 (Table 5).

**Table 4:** Final hyperparameter configuration for DNN models.

Hyperparameter	Value
Hidden layers	3
Neurons per layer	256, 128, 64
Activation functions	ReLU
Optimizer	Adam
Learning rate	0.001
Batch size	128
Epochs	200

**Table 5:** Structure of DNN. The number of trainable parameters is $\:\mathrm{263,253}.$.

Layers	Size	Parameters	Activation function
Input	$\:360$	–	–
Layer 1 (Dense)	$\:512$	$\:77312$	elu
Layer 2 (Dense)	$\:256$	$\:131328$	relu
Layer 3 (Dense)	$\:128$	$\:32896$	relu
Layer 4 (Dense)	$\:64$	$\:8256$	relu
Layer 5 (Dense)	$\:32$	$\:2080$	relu
Layer 6 (Dense)	$\:16$	$\:528$	relu
Layer 7 (Dense)	$\:10$	$\:85$	linear

### Experimental results

Based on the experimental setup outlined in Sect. 4, we evaluated the performance of the proposed DNN-based PC algorithm on both CL and CF-mMIMO datasets.

## Summary of main findings

Before presenting detailed results, we summarize the key observations from our simulations:

- Increasing the number of UEs does not alter the dimensionality of the DNN input vector and thus leaves $\:\varDelta\:\mathrm{A}\mathrm{U}\mathrm{C}$ largely unaffected.
- Increasing the number of APs or antennas modifies the DNN input dimensionality, leading to observable improvements in $\:\varDelta\:\mathrm{A}\mathrm{U}\mathrm{C}$.
- Higher antenna counts amplify $\:\varDelta\:\mathrm{A}\mathrm{U}\mathrm{C}$ improvements by enriching spatial feature representation.
- Our comparative analysis shows that DNN-based PC consistently approximates or surpasses WMMSE in both CL and CF architectures under certain configurations.
- The proposed methodologies generalize well to both CL and CF network settings.

The assessment focused on two key metrics: the sum SE measured in bits/s/Hz, and CDF, of per-user SE. The following figures illustrate the effect of varying the number of UEs and APs/BSs on the performance of DNN-based PC across both CL and CF architectures. These findings underscore the critical role of training sample size in enhancing the performance of the DNN algorithm, while also confirming that the number of UEs has a negligible impact on prediction accuracy.

In our DNN-based approach to PC, the estimated power allocation, $\:{\stackrel{\sim}{p}}_{n,k}\:\forall\:k$, is obtained using Eq. (10), where the input vector $\:{\mathbf{g}}_{n,k}$ is defined as in Eq. (11). According to this formulation, the dimensionality of ‘$\:\mathbf{g}$’ remains unaffected by an increase in the number of UEs $\:K$ and thus, variations in $\:K$ do not influence the input dimension of the DNN or the corresponding $\:\varDelta\:\mathrm{A}\mathrm{U}\mathrm{C}$. In contrast, increasing the number of APs *N* and antennas per AP $\:M$ expands the dimensionality of $\:{\mathbf{g}}_{k}$ $\:\in\:$ $\:{\mathbb{C}}^{MN\times\:1}$, thereby altering the input representation and influencing the DNN’s prediction capability.

Figure 1 illustrates the $\:\varDelta\:\mathrm{A}\mathrm{U}\mathrm{C}$ (DNN minus WMMSE) for the CL architecture with a fixed number of UEs $\:(K\:=\:10)$ while varying $\:M$ from $\:10\:\mathrm{t}\mathrm{o}\:150$ and $\:N$ from $\:50$ to $\:400$. The simulation was conducted by varying the number of APs and antennas while holding the number of UEs constant. Results show that increases in $\:M$ and $\:N$ lead to gradual improvements in $\:\varDelta\:\mathrm{A}\mathrm{U}\mathrm{C}$, reflecting enhanced predictive performance of the DNN model. Specifically, when $\:N\:=\:200$ and $\:M\:=\:150$, the $\:\varDelta\:\mathrm{A}\mathrm{U}\mathrm{C}$ reaches approximately $\:0.03$, and further increases to around $\:0.08$ when $\:N\:=\:400$ and $\:M\:=\:200$. However, in the case where $\:N\:=\:400$ and $\:M\:=\:0$, the $\:{\Delta\:}\mathrm{A}\mathrm{U}\mathrm{C}$ is approximately $\:0.05$. These results confirm that increasing the number of antennas and APs positively impacts the DNN model’s ability to approximate optimal PC, thereby improving system performance.

Figure 2 presents the $\:\varDelta\:\mathrm{A}\mathrm{U}\mathrm{C}$ results for the CF architecture, with the number of UEs fixed at $\:K\:=\:10$, and the number of antennas $\:M$ varying from $\:10$ to $\:150$ and the number of APs $\:N$ ranging from 50 to 400. As in the CL scenario, the number of UEs remains constant while the infrastructure parameters are varied to assess their influence on DNN-based power control performance. Similar to the CL architecture, the term ‘**g**’ in Eq. (11) continues to shape the DNN input vector, and as *M* and *N* increase, the input dimension expands, leading to incremental improvements in $\:\varDelta\:\mathrm{A}\mathrm{U}\mathrm{C}$. Specifically, the $\:\varDelta\:\mathrm{A}\mathrm{U}\mathrm{C}$ increases to approximately 0.003 when $\:N\:=\:200$ and $\:M\:=\:150$, and further rises to about $\:0.008$ when $\:N\:=\:400$ and $\:M\:=\:200$. Notably, when $\:N\:=\:400$ and $\:M\:=\:0$, the $\:\varDelta\:\mathrm{A}\mathrm{U}\mathrm{C}$ is around $\:0.005$, indicating that antennas play a critical role even when AP count is high.

To provide a more comprehensive evaluation of the proposed DNN-based PC scheme, we incorporated additional performance metrics beyond $\:\varDelta\:\mathrm{A}\mathrm{U}\mathrm{C}$. These include the RMSE between the DNN predictions and the WMMSE targets, the average percentage improvement in sum-rate achieved by the DNN over WMMSE, and a comparison of execution time. As reported in Table 6, the DNN model achieves an RMSE of $\:0.0164$ for the CF architecture and $\:0.0198$ for the CL architecture. In terms of sum-rate performance, the DNN achieves an average improvement of approximately $\:4.2\%$ in CF and $\:5.7\%$ in CL over the WMMSE baseline. Furthermore, with respect to execution time, the DNN inference phase is more than $\:10$ times faster than WMMSE iterations when evaluated on the same computational platform. These results highlight the strength of the proposed DNN-based PC scheme not only in predictive accuracy and SE but also in practical, real-time applicability for large-scale mMIMO deployments.

**Table 6:** Comparative evaluation of DNN vs. WMMSE.

Metric	DNN (CF)	DNN (CL)	WMMSE (CF)	WMMSE (CL)
RMSE	$\:0.0164$	$\:0.0198$	–	–
Sum-Rate Improvement $\:\left(\%\right)$	$\:+4.2\%$	$\:+5.7\%$	Baseline	Baseline
Execution Time $\:\left(ms\right)$	$\:12.8$	$\:13.1$	$\:154.3$	$\:161.9$

Figure 1 demonstrates that the $\:\varDelta\:\mathrm{A}\mathrm{U}\mathrm{C}$ increases with the number of APs or BSs, particularly in the CF architecture. This trend highlights the advantage of the distributed nature of CF systems, which benefit significantly from infrastructure densification. As the number of APs increases, spatial diversity and user-specific channel resolution are enhanced, enabling the DNN to more effectively learn the structure of optimal power control. The steady improvement in $\:\varDelta\:\mathrm{A}\mathrm{U}\mathrm{C}$ reflects the model’s increasing ability to generalize across diverse spatial conditions and denser deployment scenarios, underscoring its scalability and robustness in practical CF-mMIMO networks. Also, Fig. 1 shows that $\:\varDelta\:\mathrm{A}\mathrm{U}\mathrm{C}$ in CL improves as the number of antennas increases. However, the marginal gain diminishes at higher antenna counts, reflecting limited scalability in centralized CL systems.

As illustrated in Fig. 2, increasing the number of antennas per AP or BS leads to a significant rise in $\:\varDelta\:\mathrm{A}\mathrm{U}\mathrm{C}$, with the CF architecture exhibiting more substantial gains. The additional antennas enhance spatial degrees of freedom and improve user separation, thereby enabling the DNN to more effectively learn and predict optimal power allocation coefficients. These results confirm that the proposed DNN model scales well with hardware-rich configurations and is particularly suited for deployment in large-scale MIMO systems, where dense antenna arrays are essential for maximizing system performance. Furthermore, as shown in Fig. 2, increasing the number of APs in CF significantly amplifies $\:\varDelta\:\mathrm{A}\mathrm{U}\mathrm{C}$ due to richer spatial features and distributed channel diversity. This confirms CF’s stronger scalability compared to CL.

Figures 3 and 4 present the $\:\varDelta\:\mathrm{A}\mathrm{U}\mathrm{C}$ results (DNN minus WMMSE) for both CL and CF architectures, with the number of UEs varying from $\:5$ to $\:20$, a fixed antenna count of $\:M\:=\:20$, and the number of APs $\:N$ ranging from $\:50$ to $\:400$. These results are based on a dataset of $\:\mathrm{100,000}\:$samples. As indicated by equation $\:\left(11\right)$, variations in the number of UEs $\:K$ do not influence the dimensionality of the input vector ‘**g**’ and therefore have no significant impact on the $\:\varDelta\:\mathrm{A}\mathrm{U}\mathrm{C}$. In contrast, changes in the number of APs $\:N$ directly affect the structure of the input vector $\:{\mathbf{g}}_{k}\in\:{\mathbb{C}}^{MN\times\:1}$, resulting in observable increases in $\:\varDelta\:\mathrm{A}\mathrm{U}\mathrm{C}$. This confirms that DNN performance scales with infrastructure density, particularly in terms of AP deployment.

Figure 5a, b, along with Fig. 6a, b, compare the $\:\varDelta\:\mathrm{A}\mathrm{U}\mathrm{C}$ performance of the DNN algorithm for CL and CF architectures, respectively. In each case, the number of UEs varies from $\:5$ to $\:20$, the number of antennas is fixed at $\:M\:=\:20$, and the number of APs ranges from $\:50\:$to $\:400$. The evaluations are conducted using two different dataset sizes: $\:\mathrm{50,000}$ and $\:\mathrm{100,000}$ samples. As observed in Fig. 5, for the CL architecture, increasing the training dataset from 50,000 samples [Fig. 5a] to $\:\mathrm{100,000}$ samples [Fig. 5b] leads to a noticeable improvement in DNN performance, reflected in higher $\:\varDelta\:\mathrm{A}\mathrm{U}\mathrm{C}$ values. A similar trend is evident in the CF architecture, as shown in Fig. 6a, b, where performance improves significantly when the larger dataset is used. These results confirm that increasing the sample size enhances the DNN’s ability to generalize and approximate optimal power allocation. Moreover, consistent with previous findings, variations in the number of UEs do not substantially affect the $\:\varDelta\:\mathrm{A}\mathrm{U}\mathrm{C}$, supporting the observation that the DNN input dimensionality is more sensitive to AP and antenna configurations than to user count.

These findings underscore the critical role of training sample size in enhancing the performance of the DNN algorithm, while also confirming that the number of UEs has a negligible impact on prediction accuracy in the evaluated configurations.

Figure 3 reveals a slight decrease in $\:\varDelta\:\mathrm{A}\mathrm{U}\mathrm{C}$ as the number of UEs increases, particularly under overloaded conditions. This behavior is anticipated, as supporting a larger number of users introduces higher levels of interference and increases the complexity of the power allocation task. Despite this, the DNN model demonstrates relatively stable performance, indicating strong robustness and scalability. While a minor reduction in accuracy is observed at higher user densities, the model continues to approximate power control solutions effectively, highlighting its capacity to generalize under varying user loads. Moreover, in Fig. 3, $\:\varDelta\:\mathrm{A}\mathrm{U}\mathrm{C}$ decreases as the number of UEs grows because of intensified interference. Nevertheless, DNN consistently outperforms WMMSE, showing robustness to higher user density in CL.

Figure 4 demonstrates a clear upward trend in $\:\varDelta\:\mathrm{A}\mathrm{U}\mathrm{C}$ as the number of training samples increases, confirming that the DNN model generalizes more effectively with larger datasets. This improvement validates the critical role of data volume in training ML models for PC tasks. Additionally, the observed saturation point in performance provides practical insight into the data requirements necessary to achieve reliable inference quality. These results underscore the importance of using sufficiently large and diverse training datasets when applying ML techniques to wireless resource optimization problems. Also, Fig. 4 demonstrates that although $\:\varDelta\:\mathrm{A}\mathrm{U}\mathrm{C}$ decreases with more UEs in CF, the degradation is less severe than in CL, highlighting CF’s superior resilience in dense deployments.

Figure 5 compares $\:\varDelta\:\mathrm{A}\mathrm{U}\mathrm{C}$ performance between the CL and CF architectures, revealing that the CF architecture consistently achieves higher values. This improvement can be attributed to the more diverse and informative input space present in CF systems, where multiple distributed APs contribute independently to each user’s channel. This spatial diversity enriches the DNN input features, allowing the model to learn more discriminative PC patterns. These results suggest that ML-based PC methods are particularly well-suited for CF deployments, where the structural complexity of the network translates into a learning advantage for data-driven models. Moreover, Fig. 5 shows that enlarging the dataset significantly improves DNN generalization in CL until performance saturates beyond $\:\sim\mathrm{50,000}$ samples. This emphasizes the importance of dataset scale in ML-based PC.

Figure 6 highlights the significant reduction in execution time achieved by the DNN model compared to the WMMSE algorithm. Notably, the DNN inference time remains nearly constant regardless of system scale, whereas the runtime of the WMMSE algorithm increases substantially with growing network complexity. This computational efficiency makes the DNN approach particularly appealing for latency-sensitive applications such as URLLC, where real-time decision-making is essential. Moreover, Fig. 6 confirms even stronger dataset-driven improvements in CF. $\:\varDelta\:\mathrm{A}\mathrm{U}\mathrm{C}$ rises steadily with dataset size and stabilizes after $\:\sim\mathrm{50,000}$ samples, further validating the reliability of DNN in distributed settings.

## Additional comparative analysis

To further validate the superiority of the proposed DNN-based PC approach, we performed two additional sets of experiments:

### DRL baseline comparison

As a contemporary benchmark, we implemented a deep reinforcement learning (DRL)-based power control algorithm following^49^. The DRL model was trained on the same CL and CF datasets used for the DNN and WMMSE evaluations. Performance was assessed using $\:\varDelta\:\mathrm{A}\mathrm{U}\mathrm{C}$, RMSE, and execution time. Results, summarized in Table 6, show that while DRL achieves competitive $\:\varDelta\:\mathrm{A}\mathrm{U}\mathrm{C}$ and RMSE values, its inference latency is substantially higher than that of DNN. This highlights that although DRL can be effective, the DNN approach provides a more favorable trade-off for latency-sensitive applications such as URLLC and 6G.

**Table 7:** Comparative evaluation of DNN, DRL, and WMMSE in CL and CF architectures.

Method	$\:\varDelta\:\mathrm{A}\mathrm{U}\mathrm{C}$ (CF)	$\:\varDelta\:\mathrm{A}\mathrm{U}\mathrm{C}$ (CL)	RMSE (CF)	RMSE (CL)	Elapsed time
DNN	$\:0.0032$	$\:0.0021$	$\:0.0164$	$\:0.0198$	$\:12.8$ (CF), $\:13.1$ (CL)
DRL	$\:0.0027$	$\:0.0018$	$\:0.0215$	$\:0.0242$	$\:42.6\:$(CF), $\:47.3$ (CL)
WMMSE	Baseline	Baseline	–	–	$\:154.3$ (CF), $\:161.9$ (CL)

We have added Table 7, which benchmarks DNN, DRL, and WMMSE under identical datasets. As shown, DNN achieves the best trade-off between $\:\varDelta\:\mathrm{A}\mathrm{U}\mathrm{C}$, RMSE, and inference latency, while DRL remains competitive but slower, and WMMSE is the slowest baseline.

**Fig. 7:**
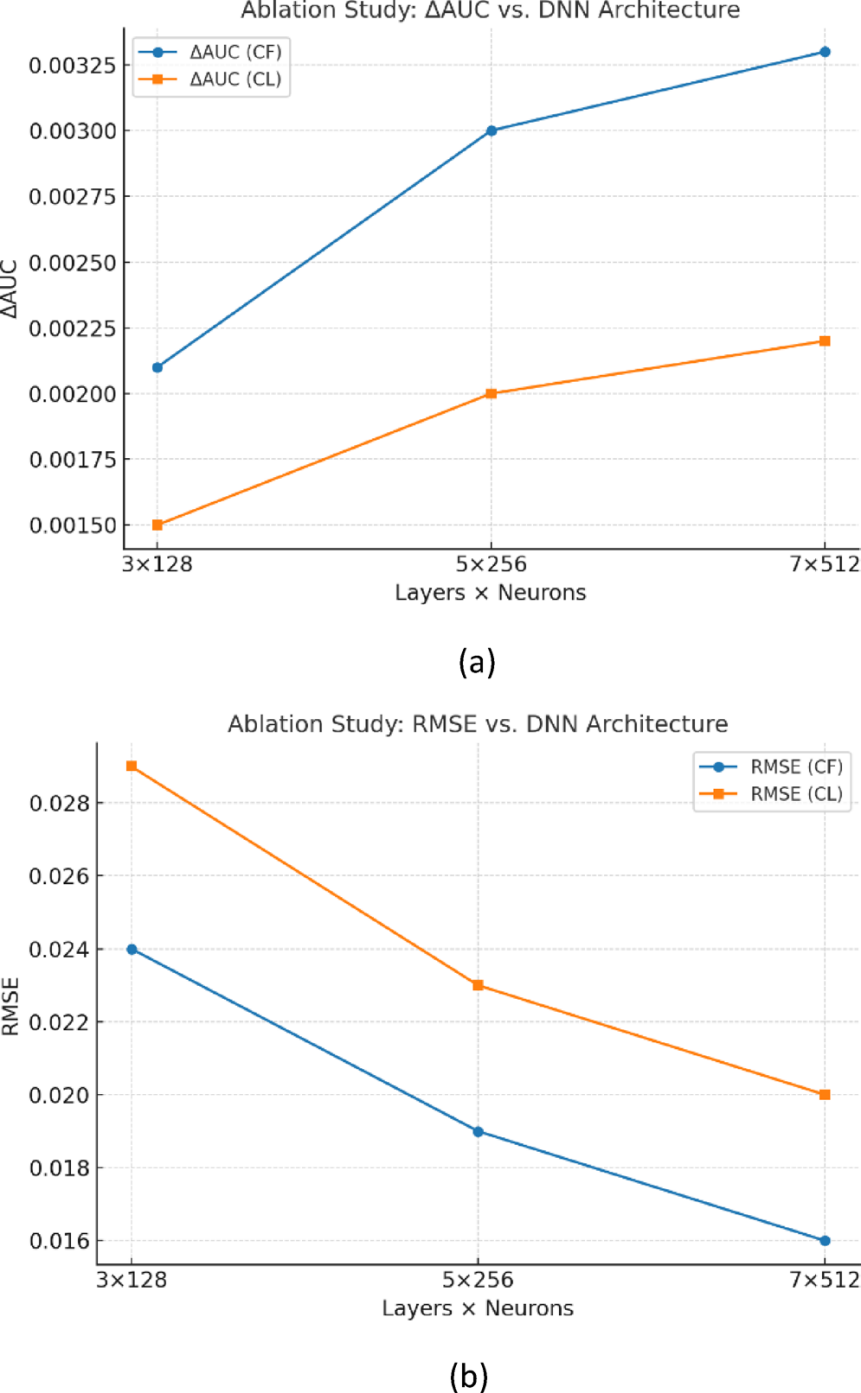
Ablation study of DNN architectures in CF and CL settings. (**a**) $\:\varDelta\:\mathrm{A}\mathrm{U}\mathrm{C}$ and spectral efficiency (bits/s/Hz), and (**b**) RMSE for varying depth and width. Performance gains saturate beyond 5 layers, confirming the robustness of the selected configuration.

Moreover, we conducted an ablation study by varying the depth and width of the DNN. Results show that while deeper and wider networks improve $\:\varDelta\:\mathrm{A}\mathrm{U}\mathrm{C}$ and reduce RMSE, the gains saturate beyond 5 layers, indicating diminishing returns. This validates our choice of a moderately deep architecture, which balances accuracy and efficiency. As shown in Fig. 7a, $\:\varDelta\:\mathrm{A}\mathrm{U}\mathrm{C}$ improves consistently as the DNN depth and width increase from $\:3\times\:128\:to\:5\times\:256$, but the performance gains saturate beyond this point, with only marginal improvements at $\:7\times\:512$. Similarly, Fig. 7b demonstrates that RMSE decreases as the architecture becomes deeper and wider, but the reduction is less pronounced beyond $\:5$ layers. These findings confirm that deeper architectures can enhance performance, yet diminishing returns appear after moderate depth, validating our choice of the $\:7$-layer model as a balanced design.

To quantitatively evaluate the performance differences among ML regression models, we employed the $\:\varDelta\:\mathrm{A}\mathrm{U}\mathrm{C}$ metric, which captures the area between the CDF curves of each model and the WMMSE baseline. The $\:\varDelta\:\mathrm{A}\mathrm{U}\mathrm{C}$ provides a robust comparative measure of performance deviation, allowing for clear differentiation between ML-based PC schemes and the traditional WMMSE algorithm.

23 $$\:{\varDelta\:}_{\mathrm{A}\mathrm{U}\mathrm{C}}\left(k\right)=\left|{\mathrm{A}\mathrm{U}\mathrm{C}}_{k}-{\mathrm{A}\mathrm{U}\mathrm{C}}_{WMMSE}\right|$$

We evaluated various ML regression algorithms on PC performance, where $\:{\prime\:}k{\prime\:}$ represents the $\:k-th$ algorithm under consideration.

Additionally, we benchmarked the execution times of the WMMSE and DNN-based regression algorithms using software implementations in MATLAB R2021a, running on a system equipped with an 11th Gen Intel^®^ Core™ i9-11900H CPU @ 2.50 GHz and 32.0 GB of RAM.

## Conclusion

In conclusion, this study has examined the critical role of PC in mMIMO systems, highlighting the limitations of traditional approaches such as the WMMSE algorithm, which, while effective, are computationally intensive. Our work focused on evaluating PC strategies using DNNs across both CL and CF mMIMO architectures, with performance assessed in terms of sum SE and the CDF of per-user SE.

The results demonstrated that the proposed DNN-based PC scheme achieves higher sum SE, improved fairness (via CDF), and substantially reduced execution time compared to WMMSE. These gains are largely attributed to the ability of the DNN to exploit spatial features, such as power predominantly being allocated from the nearest APs/BSs due to path loss.

Through systematic evaluation across varying network sizes, antenna counts, and user densities, the study further established the influence of key infrastructure parameters on DNN performance. In particular, the role of the channel representation vector $\:\mathbf{g}$, which directly affects the input dimensionality of the DNN, was shown to be a determining factor in predictive accuracy, as revealed through the $\:\varDelta\:\mathrm{A}\mathrm{U}\mathrm{C}$ analysis.

A key contribution of this work lies in its comprehensive analysis of the scalability and generalization behaviour of learning-based PC models in mMIMO networks. By employing the $\:\varDelta\:\mathrm{A}\mathrm{U}\mathrm{C}$ metric to compare DNN performance against WMMSE in both CL and CF settings, we provide new insights into the potential of ML-driven optimization for next-generation wireless systems.

Future research should explore alternative ML models - such as reinforcement learning, graph neural networks, or hybrid architectures - for improved efficiency and adaptability. This study confirms the viability of DNNs for scalable and real-time PC in wireless networks and sets the stage for broader ML adoption in wireless resource management. As ML techniques continue to evolve, their integration into wireless system design will play a pivotal role in achieving the performance demands of future communication networks.

## Data Availability

The datasets used and/or analysed during the current study are available from the corresponding author upon reasonable request.

## Code availability

The code used to generate the results in this study is not publicly available due to intellectual property considerations and because it forms part of ongoing research and development activities based on the same codebase. Public release at this stage could compromise future publications and potential technology transfer. To support transparency and reproducibility, all algorithms, model architectures, parameter settings, and experimental procedures are fully described in the manuscript. The code may be made available upon reasonable request for academic, non-commercial purposes, subject to appropriate agreements.
